# STAT3 inhibitor in oncology: a trial-based analysis of translational challenges and evolving therapeutic paradigms

**DOI:** 10.3389/fonc.2026.1802640

**Published:** 2026-05-07

**Authors:** Siyuan Yang, Lei Wang, Zhirui Chuan, Jianyun Nie

**Affiliations:** 1 Department of Breast Surgery, Peking University Cancer Hospital Yunnan Hospital, Yunnan Cancer Hospital, Third Affiliated Hospital, Kunming Medical University, Kunming, Yunnan, China; 2 Department of General Surgery, 926 Hospital of the Joint Service Support Force of the Chinese People’s Liberation Army, Kaiyuan, Yunnan, China; 3 Department of Ultrasound, Peking University Cancer Hospital Yunnan Hospital, Yunnan Cancer Hospital, Third Affiliated Hospital of Kunming Medical University, Kunming, Yunnan, China

**Keywords:** cancer therapy, clinical trials, molecular targeted therapy, STAT3 inhibitor, translational medicine

## Abstract

Signal Transducer and Activator of Transcription 3 (STAT3), a key oncogenic target, faces clinical development hurdles for its inhibitors. A systematic retrospective analysis of Trialtrove database search yielded 130 eligible oncology trials, this perspective reveals critical developmental patterns: Trial numbers peaked in 2018 but declined post-2019, reflecting Phase III attrition due to efficacy-safety imbalances and enrollment challenges. The evolving landscape demonstrates a paradigm shift from conventional Src homology 2 (SH2) domain inhibitors toward next-generation strategies targeting the coiled-coil domain, mitochondrial STAT3, and biomarker-guided patient stratification. Realizing STAT3’s therapeutic potential requires biomarker-guided patient selection, innovative inhibitors for enhanced specificity, and optimized trial strategies to bridge preclinical-clinical gaps. Learning from past outcomes and prioritizing predictive biomarkers are critical for advancing STAT3-targeted cancer therapy.

## 1 Introduction

Signal transducer and activator of transcription 3 (STAT3) is essential for cell growth, differentiation, survival, and immune responses (1, 2). However, its overexpression and aberrant activation contribute to cancer proliferation, metastasis, and survival, positioning STAT3 as a key oncogene and a prime therapeutic target (3, 4).

STAT3 consists of six conserved domains, each contributing distinct functional roles: the N-terminal domain (NTD), coiled-coil domain (CCD), DNA-binding domain (DBD), linker domain (LD), Src homology 2 (SH2) domain, and C-terminal domain (5, 6). The NTD facilitates tetramerization of phosphorylated STAT3 dimers, dimerization of unphosphorylated STAT3, and protein–protein interactions essential for DNA binding, nuclear accumulation, and gene regulation (7). The CCD participates in early signaling events, including receptor recruitment, tyrosine phosphorylation, dimerization, and DNA binding (8). The DBD recognizes specific DNA sequences to promote target gene expression (9), while the SH2 domain is a highly conserved region critical for phosphorylation-dependent dimerization (10). The three-dimensional architecture of these domains reveals binding sites distinct from the canonical phosphotyrosine-binding pocket, offering opportunities to enhance inhibitor specificity.

These structural features have guided the development of STAT3-targeted strategies, with notable progress achieved through domain-specific approaches (11–13). The SH2 domain, in particular, has emerged as the primary focus of inhibitor development due to its essential role in STAT3 dimerization—a prerequisite for nuclear translocation and DNA binding. However, the relatively shallow and featureless nature of the phosphotyrosine-binding pocket poses a significant challenge for developing small-molecule inhibitors with high affinity and selectivity (14). Moreover, the large size of the protein–protein interaction interfaces and the involvement of STAT3 in non-canonical functions continue to impede progress in both preclinical and clinical trials of STAT3 inhibitors (15).

Despite these advances, detailed investigations and systematic analyses of STAT3-targeted clinical trials remain limited. While numerous inhibitors have entered clinical evaluation, the field lacks a comprehensive synthesis of trial outcomes, including the structural basis of inhibitor activity, off-target effects, and the factors underlying clinical success or failure. A comprehensive review of past and ongoing clinical trials targeting STAT3 is necessary to distill lessons from prior successes and failures. Here, we present a comprehensive analysis of the clinical trial database to offer a panoramic perspective of clinical research targeting STAT3 in cancer, aiming to inform the future research directions.

## 2 Methods

### 2.1 Data source and search strategy

In this study, the Trialtrove database (https://clinicalintelligence.citeline.com/) was used for a comprehensive search. The search was conducted from database inception up to October 14, 2025. The compound search terms included: [(Mechanism of Action: STAT transcription factor 3 inhibitor)] AND [(Therapeutic Area is Oncology)].

### 2.2 Study selection and data extraction

Two individual investigators independently reviewed and rechecked the source data to ensure the accuracy of the relevant conclusions by the research team. After the exclusion of studies without detailed information and not included STAT3, 130 eligible trials were identified eventually.

## 3 Results

**Figure 1:**
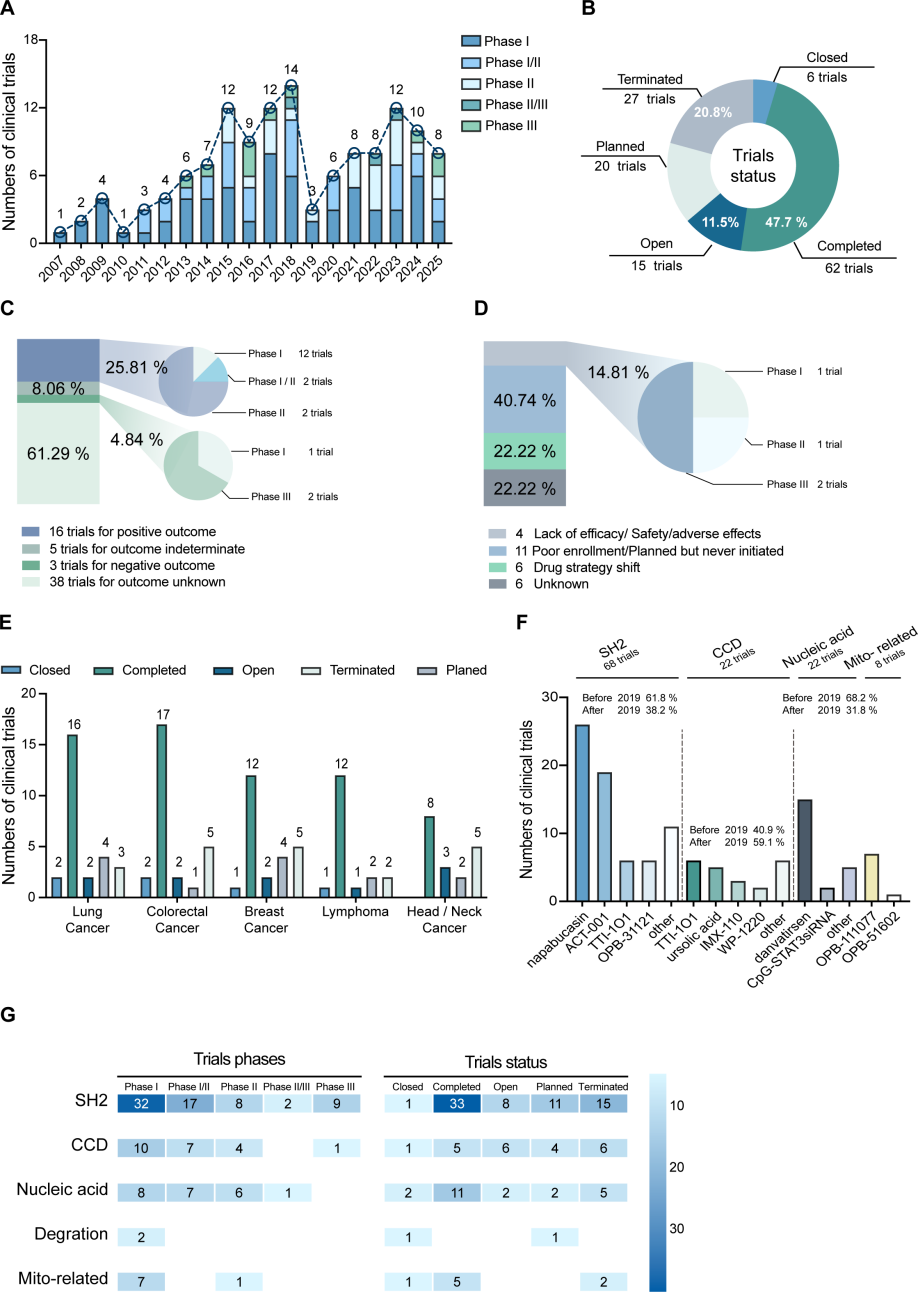
Landscape of clinical trials of STAT3 inhibitors. **(A)** Temporal Trends in clinical trials of STAT3 inhibitors; **(B)** Distribution of STAT3 inhibitors clinical trials by Trial status. **(C)** The outcome of completed trials; **(D)** The outcome of terminated trials; **(E)** Trial distribution of STAT3 inhibitors in TOP5 cancer based on trial status**. (F)** Targets distribution of STAT3 inhibitors; **(G)** Trends in STAT3 inhibitors targets by trial phase and status.

Analysis of the data revealed that clinical trials involving STAT3 inhibitors have undergone significant fluctuations. In contrast to the overall increasing trend observed across all oncology clinical trials during the same period, the number of studies focusing on STAT3 inhibitors surged markedly from 2010, peaking in 2018, before experiencing a sharp decline in 2019. Following this decline, the number of clinical trials related to STAT3 inhibitors gradually increased once more (**Figure 1A**). This trajectory suggests that STAT3, recognized as a highly promising target for anti-tumor therapies, garnered considerable attention during early stage. However, challenges related to efficacy and safety led to a temporary slowdown in new trial initiations, from which the field has now gradually recovered. Consistent with this trend of recovery, the current trial landscape comprises 62 completed studies (47.7%) and 15 ongoing trials (11.5%), underscoring the potential of this strategic approach. Nevertheless, the field has also experienced 27 terminations (20.8%) (**Figure 1B**), reflecting the inherent challenges that have shaped its developmental trajectory.

To investigate the reasons for this temporary slowdown, we observed several interesting phenomena. First, among the completed groups that yielded positive outcome (16/62, 25.8%), all clinical trials were in the early stages, with Phase I trials comprising the largest proportion. Second, within the completed groups that positive endpoint(s) had not met (3/62, 4.84%), Phase III trials were particularly representative (**Figure 1C**). This suggests that the development of STAT3 inhibitors has reached a “phased” maturity in preclinical phase. In terms of termination reasons, poor enrollment (11/27, 40.74%) and pipeline reprioritization (6/27, 22.22%) were the most frequent, while lack of efficacy (e.g., unsatisfactory disease control rate) and safety issues (e.g., intolerable lactic acid and metabolic acidosis) accounted for 4 terminations (14.81%), primarily affecting Phase III trials (**Figure 1D**). Notably, SH2 domain inhibitors—predominantly Napabucasin—accounted for 15 of the terminated trials (15/27, 55.6%), with failures largely attributed to lack of efficacy in Phase III studies. Therefore, the core challenge in advancing STAT3 inhibitor development lies in identifying more optimal targets to bridge the gap between preclinical anti-tumor efficacy and clinical benefit while minimizing the incidence of adverse events.

Beyond precise target selection, researchers must also consider additional factors, chief among them cancer type. Our analysis revealed that lung cancer (27), colon cancer (27), breast cancer (24), lymphoma (18), and head/neck cancer (18) emerged as the top five indications (**Figure 1E**). This distribution pattern reflects a focus on solid tumors such as lung, colon, and breast cancers, as well as non-solid tumors like lymphoma, which may be attributable to the differential expression levels and activation patterns of STAT3 across these cancers.

Therapeutic strategies targeting specific domains within the STAT3 protein show promise in addressing pathological functions. Firstly, blocking the SH2 domain can prevent the dimerization and transcriptional activity of STAT3, thereby inhibiting tumor growth and metastasis. Therefore, most of the existing STAT3 inhibitors are concentrated in the SRC homology 2 domain (SH2) (68/130, 52.3%), followed by the coiled-coil domain (CCD) in which competitive inhibition exerts regulatory functions on tyrosine phosphorylation and dimerization in the early stage of STAT3 signal transduction (22/130, 16.9%). Given that aberrant overexpression of STAT3 serves as a critical mediator of pro-malignant phenotypes, trials targeting STAT3 mRNA translation inhibition comprised 16.9% of the cohort (22/130 trials). Notably, however, only 2 trials (1.5%) specifically focused on promoting STAT3 protein degradation by PROTAC. Furthermore, mitochondrial function and mitochondrial STAT3 (mSTAT3) plays a crucial role in oncogene-induced signaling and cancer cell metabolism, establishing them as valuable therapeutic targets (2). This recognition has contributed to an increased proportion of clinical trials focusing on these pathways, which now account for 6.1% (8/130). In addition, inhibitors specifically targeting the coiled-coil domain (CCD) have dominated clinical investigation since 2019, comprising 59.1% (13/22) of post-2019 trials. These trends underscore the evolving paradigm in STAT3 inhibitor design, exemplified by next-generation candidates like TTI-101 that employ dual-target strategies. Such approaches aim to achieve enhanced specificity and efficacy through simultaneous engagement of distinct STAT3 domains, thereby optimizing tumoricidal activity while minimizing off-target effects.

Inhibitors targeting the SH2 domain (32/68, 47.1%), the CCD (10/22, 45.5%), and STAT3 mRNA translation suppression (8/22, 36.4%) are prevalent in Phase I clinical trials (**Figure 1F**). Despite this, rigorous validation efforts continue for the maturation of STAT3 inhibitor development. Ongoing open-label and preparatory trials aim to ensure robust efficacy and safety assessment. This translational phase, marked by sustained investigational activity, signifies advancing translational momentum and enhances confidence in STAT3-targeted inhibitors.

## 4 Discussion

The temporal trajectory of STAT3 inhibitor clinical trials shows a surge post-2010, peaking in 2018, aligned with preclinical breakthroughs establishing STAT3 as a cancer regulator. A 2019 decline and subsequent recovery reveal a “valley of death” period where early enthusiasm clashed with clinical realities—a pattern mirrored in oncology drug development due to challenges in balancing efficacy and target validation.

Trial status analysis provides granular insights into developmental bottlenecks. Completed studies (47.7%) demonstrate feasibility but reveal a concerning Phase III failure rate (2/3 trials). Through our analysis, in a previously published Phase III clinical trial of colorectal cancer (NCT01830621), the OS of the Napabucasin group was 4.4 months compared with 4.8 months in the placebo group, which failed to meet expectations (16). Therefore, the insufficient transformation of preclinical effects has seriously hindered the development of STAT3 inhibition. Terminated trials (20.8%) further highlight systemic challenges: 40.7% failed due to poor enrollment, reflecting trial design issues, while 22.2% succumbed to pipeline reprioritization, suggesting strategic shifts in pharmaceutical portfolios. It is comforting that the 11.5% ongoing trials, coupled with burgeoning activity in novel mechanisms, indicate enduring industry and academic commitment, underscoring STAT3’s enduring therapeutic allure.

Strategic priorities for next-generation inhibitors thus coalesce around three axes: cancer type refinement, target optimization, and mechanism innovation. Lung, colorectal, breast cancers, and lymphomas dominate current indications (more than 80% of trials), driven by STAT3’s differential expression/activation signatures (17–20) and their disproportionate global burden (21). However, even within the same inhibitor, indication selection demands precision. For instance, BBI608 (22) showed efficacy in metastatic pancreatic cancer (NCT02231723) but failed in unselected colorectal cancer—though pSTAT3-positive subsets derived OS benefit (5.1 vs. 3.0 months). This underscores the imperative for biomarker-guided patient stratification (16). Therefore, for the top five indications which account for 87.7% of the total trials, the stratification for biomarker-guided patient is imperative.

Mechanistic evolution is equally striking. While SH2 domain inhibitors remain prevalent (52.3%), emerging CCD-targeted (16.9%) and mitochondrial-targeted trials (6.1%) signal a paradigm shift from single-domain suppression to combinatorial strategies. S3I-201, an early SH2 inhibitor, exemplifies first-generation limitations: its shallow binding pocket and weak affinity precipitated off-target alkylation (23). In contrast, TTI-101’s dual inhibition—achieved through SH2 domian blockade and TAD modulation based on Tyr705 phosphorylation blockade —demonstrates enhanced specificity and therapeutic index, as validated in preclinical models and Phase I trials (NCT03195699) (24, 25). The shift in the dominant position of CCD inhibitors after 2019 (59.1% of the trials) reflects their potential in early efficacy. For mitochondrial function and mSTAT3, OPB-111077 can directly inhibit the activity of mitochondrial respiratory chain complex I (OXPHOS) (26), whereas OPB-51602 was proven to directly interfere with mSTAT3. However, during this process, we noticed that both can directly bind to the SH2 domain, suggesting a dual mechanism of action that enhance the anti-tumor effect (27). Additionally, diffuse large B-cell lymphoma showed partial efficacy to OPB-111077, while unselected solid tumors only showed limited efficacy (26). Therefore, the therapeutic benefit may depend on the degree of tumor dependence on mitochondria function or the expression level of mSTAT3.These methods aim to enhance specificity by targeting the key sites of the domain and alleviate the off-target effects that have plagued the first-generation inhibitors (**Table 1**).

**Table 1:** Key STAT3 inhibitors discussed in the clinical landscape.

Inhibitor	Mechanism of action	Key findings	References
Napabucasin(BBI608)	SH2 domain inhibitor	Phase III trial (NCT01830621) in refractory advanced colorectal cancer failed to meet OS endpoint (4.4 vs 4.8 months); pSTAT3- positive subsets derived OS benefit (5. I vs 3.0 months)	(16)
S3I-201	SH2 domain inhibitor	Demonstrated selective inhibition of STAT3 activation and DNA-binding activity	(23)
TTI-101 (C188-9)	Dual inhibitor: SH2 domain blockade + TAD modulation based on Tyr705 phosphoryla1ion blockade	Phase I trial (NCT03195699) showed well-tolerated safety profile; confirmed partial responses in HCC, ovarian, and gastric cancers	(24, 25)
OPB-111077	SH2 STAT3 inhibitor; directly inhibits mitochondrial respiratory chain complex I	Demonstrated partial efficacy in diffuse large B-cell lymphoma but limited activity in unselected solid tumors.	(26)
OPB-51602	Dual inhibitor: directly interferes with mSTAT3 +binds to SH2 domain	Interferes with mitochondrial activity and mSTAT3, leading to a synthetic lethality effect in glucose-depleted cancer cells.	(27)

Critical challenges persist in bridging preclinical efficacy to clinical utility. Phase I successes (25.8% positive outcomes) contrast with Phase III attrition, underscoring the need for robust biomarkers (e.g., pSTAT3) and adaptive trial designs. The underrepresentation of PROTAC strategies (1.5%) highlights untapped potential in protein degradation. Although there is evidence suggesting that STAT3 PROTAC can achieve complete degradation of STAT3 in both xenograft tumor tissues and normal mouse tissues while maintaining good tolerance (28), further strategies such as tumor-specific E3 ligase recruitment are still needed to achieve tumor-selective degradation.

## 5 Conclusion

In conclusion, the clinical translation of STAT3 inhibitors faces challenges in converting preclinical validation into successful patient therapies. Despite being a well-established oncology target, high failure rates in late-stage trials hinder progress. Key strategies for future success include refining patient selection with predictive biomarkers, developing next-generation inhibitors targeting novel mechanisms, and utilizing innovative therapeutic approaches like STAT3-specific decoy oligonucleotides (STAT3-decoy ODN) (29, 30). Optimizing clinical trial design is essential to overcome translational hurdles and fully realize the promise of STAT3 inhibition in cancer treatment.

## Data Availability

The original contributions presented in the study are included in the **Supplementary Material**. Further inquiries can be directed to the corresponding authors.
